# Evaluation of antioxidant effects of crocin on sperm quality in cyclophosphamide treated adult mice

**Published:** 2014

**Authors:** Zahra Bakhtiary, Rasoul Shahrooz, Abbas Ahmadi, Leila Zarei

**Affiliations:** 1 *Department of Basic Sciences, Faculty of Veterinary Medicine, Urmia University, Urmia, Iran; *; 2 *Maternal and Childhood Obesity Research Center, Urmia University of Medical Sciences, Urmia, Iran.*

**Keywords:** Crocin, Cyclophosphamide, Mice, Sperm quality

## Abstract

Cyclophosphamide (CP) is one of the anti-neoplastic drugs. Despite its numerous clinical applications, it has devastating effects on the testicles and declines the sperm quality in treated patients. This study was aimed to investigate the protective effect of crocin in improving the toxicity induced by CP in reproductive system. In this study, 24 male adult mice (6 to 8 weeks) were randomly divided into three groups, control group received normal saline (0.1 mL, IP, daily), the CP group received CP (15 mg kg^-1^, IP, weekly) and the CP + crocin group received CP along with crocin (200 mg kg^-1^, IP, daily). After 35 days of treatment, animals were sacrificed. The samples of epididymis in human tubal fluid medium incubated for 30 min in 5% CO_2_ for flotation of sperm. Sperm were obtained from caudal epididymis using dissecting method. Then, the parameters of sperm quality including sperm count, motility, viability, DNA damage, nuclear maturation, and sperm morphology were evaluated. In CP group, the sperm count, motility, viability, nuclear maturation and sperm morphology were significantly decreased compared to control group (*p* < 0.05) and in the CP + crocin group all of these parameters significantly increased compared to CP group (*p* < 0.05). The percentage of sperm with DNA damage in the CP group significantly increased compared to other groups (*p* < 0.05). The results of this study indicated that the crocin was able to suppress free radicals and enhance the quality of sperm in CP treated animals.

## Introduction

One of the current drugs used in chemotherapy is cyclophosphamide (CP), in addition to its beneficial therapeutic effects, by disrupting of antioxidant system in tissues and increasing the oxidative stress,^1^^-^^3^ decreases fertility in treated patients.^4^^,^^5^ According to previous studies, CP may negatively affect testis tissue and epididymis.^6^ It can also affect the sperms generation in testes and sperm maturation in the epididymis.^7^ Patients treated with this drug for four months or more have experienced varying states of oligospermia and azoospermia.^8^ Drugs with alkylating properties have the most deleterious effects on the testes.^9^ One of the major side effect of CP as an alkylating agent and a cytotoxic substance^10^^,^^11^ is impairment of sperm quality and fertility.^10^^,^^11^ Acrolein, a toxic metabolite of CP, interferes with the body's antioxidant system and a high level of reactive oxygen species (ROS) is produced.^12^^,^^13^ Compounds with anti-oxidant properties enable the body to fight against conditions caused by ROS or free radicals.^2^^,^^14^ Thus, prescription of antioxidant compounds during chemo-therapy in order to reduce oxidative stress due to CP and increase fertility parameters seems necessary. Enzymatic and non-enzymatic antioxidants serve as an important biological defense against environmental pollutants. Various enzymatic and non-enzymatic antioxidants as a stress biomarker in liver and kidney of rat have investigated. The antioxidant enzymes included superoxide dismutase, catalase, glutathione reductase, glutathione-S-transferase, and glutathione peroxidase have been studied.^15^ Recent pharmacological studies have demonstrated that saffron extracts as non-enzymatic antioxidants have antitumor effects, radical scavenger properties and hypolipemic effects. Among the constituents of saffron extract, crocin is mainly responsible for these pharmacological activities.^16^

Saffron cultivated in Iran and other countries such as India and Greece, has been used in traditional medicine as an anti-cough and anti-sputum.^17^^,^^18^ Compounds in saffron have numerous health benefits, such as anti-nociceptive, anti-inflammatory,^19^ anti-convulsants,^20^ antidepressants in animals,^21^^,^^22^ and human.^23^ One of the main ingredients of saffron is crocin,^24^ and it is a water-soluble carotenoid unit used in the treatment of patients with neurological degeneration and loss of memory.^16^ Pharmacological studies have shown this substance is a free radicals scavenger.^24^ Whereas, the effect of crocin on the reproductive potential of patients treated with CP has not been studied, this research was aimed to evaluate the protective effect of crocin on semen parameters such as average number, motility status, viability, DNA damage and nuclear maturation of sperm to be done. 

## Materials and Methods

**Animals and treatment groups.** In this study, 24 adult male mice of NMRI rat aged 8 to 12 weeks were kept up in standard conditions of temperature 22 ± 2 ˚C, 30 to 60 % humidity and the light period of 14 hr light and 10 hr of darkness. Animals were randomly divided into three groups: Control, CP and CP + crocin groups, as below :

1. Control group received normal saline (0.1 mL, daily, IP).
2. Cyclophosphamide group received only CP (Baxter, Frankfurt, Germany; 15 mg kg^-1^; weekly, IP).
3. Cyclophosphamide + crocin group received crocin (Sigma-Aldrich, St. Louis, USA; 200 mg kg^-1^, daily, IP) in addition to CP (the same dose).

**Sperm sampling.** After 35 days of treatment, mice were anesthetized and euthanized with 25 mg kg^-1 ^ketamine (Alfasan, Woerden, The Netherlands) and the abdominal skin was sterilized with 70% ethanol. After euthanasia by 100 mg kg^-1 ^ketamine (Alfasan, Woerden, The Netherlands), the epididymis was removed and transferred to Petri dish 6 cm containing medium 1 mL of human tubal fluid medium (HTF; Sigma-Aldrich, St. Louis, USA) and 4 mL^-1 ^of bovine serum albumin (BSA; Sigma, St. Louis, USA) that previously its temperature was balanced by incubator (5% CO_2_, 37 ˚C) and then, by making a few incision in the epididymis and 30 min incubation at 37 ˚C in 5% CO_2_, spermatozoa released from epididymis.

**S** **perm count** **. **For sperm counting at 1:20 dilution from sperm samples were prepared. For this purpose 10 µL of the sperms were added to 190 µL of distilled water, and then 10 µL of the dilated sperm was dropped on a Neubauer slide and the average number of sperms were counted.^25^

**Sperm motility. **The medium (10 mL) of containing sperm was placed on the Neubauer slide and under a light microscope with a magnification of 20× the percentage of sperm motility was evaluated. 

**Sperms viability.** Semen sample (20 µL) of was placed onto a clean slide and then 20 µL of eosin solution was added to it, after 30 sec, 20 µL of nigrosin solution was added. Then, from the desired solution, smear was prepared and after drying slides and using a light microscope with 40× magnification percentage of alive sperm (colorless) and dead sperm (red color in head) were determined.^26^

**DNA strand damage.** The semen samples were washed three times with phosphate buffered saline (PBS) and after removal of the supernatant, the sediment was achieved by using PBS to a final concentration. Then, smears were prepared from the medium containing sperm and after drying in a laboratory environment, for 30 min, was placed into in acetone - ethanol (1:1) container.

After drying in the air, coverslips were placed in acridine orange staining solution for 7 min and after the final drying in a dark place, the slides were examined using an immunofluorescence microscope (Model 466300; Carl Ziss, Jena, Germany) with 100× objective magnification and the results were reported as percentage.^27^^,^^28^

**Sperm chromatin condensation.** Similar to the above procedure, after fixing slides in ethanol - acetone solution and drying in air, they were placed in aniline blue staining solution for 7 min and after drying in air, were observed by light microscope in magnification of 100×. In this staining method, immature sperms with the proportion of histone protein in the nucleus become the color of dark blue gray and mature sperms showed pale.^29^

**Sperm morphology** **.** In this process, two staining methods, aniline blue and eosin-nigrosin were used. Sperms that appeared abnormal by aniline blue staining were counted and results were expressed as percentage and using eosin-nigrosin staining, spermatozoa containing cytoplasmic debris were counted as immature sperms.^30^

**Statistical analysis. **The data were analyzed by SPSS (Version 20; SPSS Inc., Chicago, USA) and two way ANOVA and Bonferroni test were used. A *p*-value less than 0.05 was considered significant. Data are presented as mean ± standard error (SE).

## Results

**Sperm count.** The results showed a significant difference between average number of sperms in CP group compared to control and CP + crocin groups (*p* < 0.05), (Table 1). 

**Sperm motility.** The results for mean percentage of motile sperms in the studied groups indicated a significant decrease of this parameter in the CP group compared to the control and CP + crocin groups (*p* < 0.05), (Table 1). 

**Sperm viability.** Results of alive sperms using eosin-nigrosin staining indicated a significant decrease of sperm viability in CP group compared with the other groups and a significant increase in CP + crocin group compared to the other groups was found (*p* < 0.05), (Table 1).

**DNA integrity.** Sperms observed with green nuclei were normal, and sperms with yellow, orange to red nucleus depending on the intensive of damage, recognized as sperms with DNA damage (Fig. 2). A significant increase in the mean percentage of sperm with damaged of DNA in CP group compared with the control group and CP + crocin group were observed (*p* < 0.05), (Table 2).

**Fig. 1:**
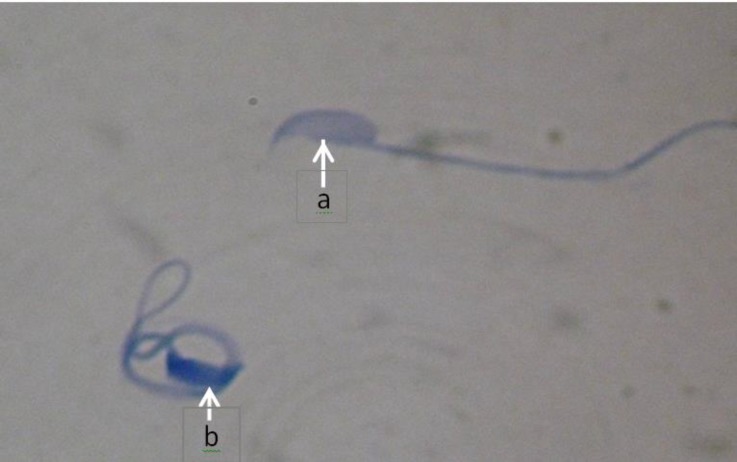
Chromatin condensation. **a.** Sperms with mature nucleus are stained with pale blue head; **b.** Immature nucleus of sperm is stained with dark blue head, (Aniline blue, 1000×).

**Sperm chromatin condensation. **After aniline-blue staining the mean numbers of immature sperms in all groups were calculated (Fig. 1). The mean percentage of immature sperms in CP group compared to control and CP + crocin groups showed a significant increase (*p* < 0.05), (Table 2).

**Sperm morphology.** In this study, the percentage of sperm with normal morphology was calculated. Results showed a significant decrease of this parameter in CP group compared with the other groups, and crocin in the CP + crocin group significantly increased this parameter (*p* <0.05), (Table 1). 

**Table 1:** Different parameters of sperm quality. Data are presented as mean ± SE

***Groups***	**Sperm count ** **(×10** ^6 ^ **per ** **mL)**	**Motility** ** (%)**	**Viability** ** (** **%)**	**Normal sperms (%)**
**Control**	32.00 ± 0.65	61.00 ± 2.12	68.50 ± 0.64	92.25 ± 0.85
**Cyclophosphamide**	15.87 ± 1.28^a^	35.77 ± 2.75^a^	40.00 ± 3.03^a^	61.75 ± 0.85^a^
**Cyclophosphamide + crocin**	28.75 ± 2.18^b^	51.50 ± 3.12^b^	53.25 ± 1.89^a^^b^	77.00 ± 3.29^a^^b^

a, b indicate significant difference with control and cyclophosphamide groups, respectively (*p *< 0.05).

**Table 2:** Percentage of sperm chromatin condensation and DNA disintegrity in different groups. Data are presented as mean ± SE

**Groups**	**Chromatin condensation** *	**DNA integrity of sperms** **
**Control**	2.00 ± 0.71	2.00 ± 0.41
**Cyclophosphamide**	41.75 ± 3.75^a^	36.00 ± 2.79^a^
**Cyclophosphamide + crocin **	18.50 ± 1.55^a^^b^	10.50 ± 1.19^a^^b^

ab indicate significant difference with control and cyclophosphamide groups respectively (*p *< 0.05).

* Aniline blue positive and

** Acridine orange positive.

**Fig. 2:**
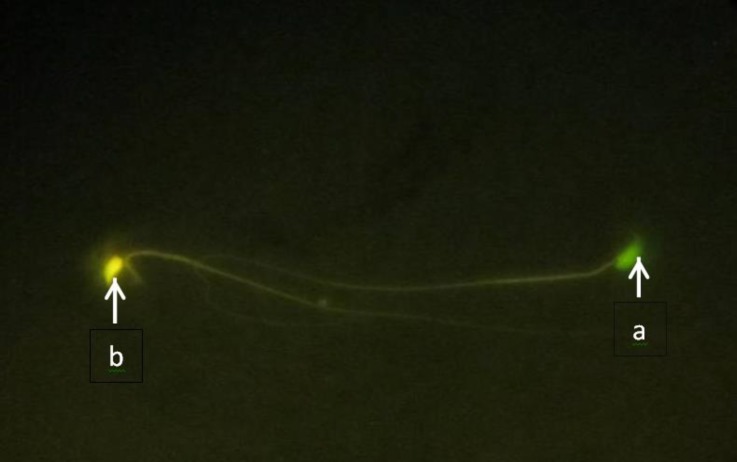
Fluorescent image of spermatozoa. **a.** Intact DNA marked with light green appearance; **b.** Sperm with damaged DNA presented with light yellow appearance, (Acridine orange, 1000×).

## Discussion

Although CP is known for its anti-neoplastic properties, there are several reports of its cytotoxic impacts via enhancing the oxidative stress. In this regard, Lear *et al.*, showed that chronic administration of CP resulted in an increased lipid peroxidation in rats.^31^^,^^32^ On the other hand, Crocin is used for treatment of dyslipidemia and atherosclerosis.^33^ The antioxidant agents of crocin result in considerable inhibition of free radical generation in different tissues.^24^ Considering the CP’s impact on antioxidant status, we hypothesized that crocin with antioxidant properties could inhibit the CP-dependent damages. In present study we showed that CP significantly reduced the sperm count, motility and viability. Accordingly, a remarkable elevation in sperm abnormality was observed in CP-treated animals. Meanwhile, administration of crocin as a potent anti-oxidant compound down-regulated the CP-induced damage and enhanced the sperm motility and viability. 

Our preliminary data showed that CP significantly decreased sperm count and motility. Reduction of sperm count, mainly attributed to beginning of testicular tissue damage. While decreased sperm motility largely depended on oxidative stress-induced derangements. Accordingly, the first sign of increased ROS content is remarkable reduction in sperm motility.^34^^-^^39^ The increased levels of ROS considerably influences sperm enzymatic content, and increases phospholipids peroxidation, which ultimately reduces fluidity of cell membrane and sperm motility.^40^^,^^41^ However, administration of crocin resulted in significant enhancement in sperm count as well as motility. Therefore, we could suggest that crocin might up-regulated the antioxidant status, which in turn elevated CP-induced damage in testicular tissue and up-regulated the CP-reduced sperm motility. 

Observations demonstrated that CP-treated animals exhibited a remarkable increase in sperm abnormality. In order to understand how CP induced this impairment, one should note that in a physiologic conditions during the maturation process, a part of the sperm cytoplasm remove as residual bodies by Sertoli cells, otherwise the cyto-plasmic droplets will be remain at the middle piece of sperm. Thus, the sperms with cytoplasmic droplets did not complete their maturation process.^42^^-^^44^ Therefore, it is logical to hypothesis that CP exerts its degenerative impact partly via influencing the Sertoli cells physiologic function, which could be partly attributed to CP-induced oxidative stress. On the other hand, the crocin-received animals showed a significantly reduction in percentage of abnormal sperms. Thus, we can assume that crocin provokes the Sertoli cells physiologic activity partly by enhancing the antioxidant status. 

Reportedly, there is a positive correlation between increased ROS generation and degenerative changes in all nucleic bases, including removal and unpairing of complementary bases, deformation and changes in cross-linking of DNA and chromosomal rearrangement.^45^^,^^46^ As a result, these impairments lead to a severe DNA damage at sperm level. In close relation with these findings, our data showed that CP increased sperm DNA damage and crocin significantly decreased the percentage of sperms with DNA disintegrity. However, the protective role of chromatin condensation against free radicals should not be forgotten. Accordingly, most of the DNA damage happens during intermediate stages of spermiogenesis when replacement of protamine-histone occurs^47^. Moreover, CP affects the protamine-DNA binding processes and therefore results in alkilation of protamine. In correlation with these reports, CP-treated animals showed a remarkable reduction in percentage of sperms with condensed chromatin. Contrarily, crocin up-regulated protamination process. Thus, we can hypothesis that crocin protected the DNA content of the sperms via provoking the protamination. 

The sperm plasma membrane integrity and controlled lipid peroxidation play important role in sperm viability. Accordingly, increased lipid peroxidation associated with continuing oxidative stress lead to severe damage at plasma membrane level.^48^^,^^49^ As it is mentioned previously CP results in severe lipid peroxidation by elevating the oxidative stress.^32^^,^^33^ Light microscopic evaluation of sperm viability showed that CP-treated animals exhibited significantly higher percentage of death sperms comparing to crocin-received group. Thus, we can suggest that crocin reduced CP-induced mortality partly by promoting antioxidant status and reducing lipid peroxidation. 

In conclusion, our data suggested that crocin as an antioxidant compound was able to inhibit the CP-induced damage at sperm level. Thus, crocin can be co-administrated with CP in order to protect sperms against CP-dependent derangements. 

## References

[1] Haque R, Bin-Hafeez B, Ahmad I (2001). Protective effects of Emblica officinalis Gaertn In cyclophosphamide-treated mice. Hum Exp Toxicol.

[2] Das UB, Mallick M, Debnath JM (2002). Protective effect of ascorbic acid on cyclophosphamide- induced testicular gametogenic and androgenic disorders in male rats. Asian J Androl.

[3] Ghosh D, Das UB, Ghosh S (2002). Testicular gametogenic and steroidogenic activities in cyclophosphamide treated rat: A correlative study with testicular oxidative stress. Drug Chem Toxicol.

[4] Howell SJ, Shalet SM (1998). Gonadal damage from chemotherapy and radiotherapy. Endocrinol Metab Clin North Am.

[5] Chapman RM (1983). Gonadal injury resulting from chemotherapy. Am J Ind Med.

[6] Trasler JM, Hermo L, Robaire B (1988). Morphological changes in the testis and epididymis of rats treated with cyclophosphamide: A quantitative approach. Biol Reprod.

[7] Trasler JM, Hales BF, Robaire B (1986). Chronic low dose cyclophosphamide treatment of adult male rats: Effect on fertility, pregnancy outcome and progeny. Biol Reprod.

[8] Qureshi MS, Pennington JH, Goldsmith HJ (1972). Cyclophosphamide therapy and sterility. Lancet.

[9] Howell SJ, Shalet SM (2005). Spermatogenesis after cancer treatment: damage and recovery. J Natl Cancer Inst Monogr..

[10] Dollery C, Dollery C (1999). Cyclophosphamide. Therapeutic drugs.

[11] Goldberg MA, Antin JH, Guinan EC (1986). Cyclophosphamide cardiotoxicity: An analysis of doing as a risk factor. Blood.

[12] Arumugam N, Silvakumar V, Thanislass J (1997). Effects of acrolein on rat liver antioxidant defense system. Indian J Exp Biol.

[13] Mythili Y, Sudharsan PT, Sulvakumar E (2004). Protective effect of DL-α-lipoic acid on cyclophosphamide induced oxidative cardiac injury. Chem Biol Interact.

[14] Ghosh D, Das UB, Misro M (2002). Protective role of alpha-tocopherol-succinate (provitamin-E) in cyclophosphamide induced testicular gametogenic and steroidogenic disorders: A correlative approach to oxidative stress. Free Radic Res.

[15] Tabrez S, Ahmad M (2011). Some enzymatic/nonenzymatic antioxidants as potential stress biomarkers of tri-chloroethylene, heavy metal mixture, and ethyl alcohol in rat tissues. Environ Toxicol.

[16] Abe K, Saito H (2000). Effects of saffron extract and its constituent crocin on learning behaviour and long-term potentiation. Phytother Res.

[17] Duke JA, Ayensu ES (1985). Medicinal plants of China.

[18] Zargari A (1990). Medicinal plants.

[19] Hosseinzadeh H, Younesi H (2002). Antinociceptive and anti-inflammatory effects of Crocus sativusL stigma and Petal extracts in mice. BMC Pharmacol.

[20] Hosseinzadeh H, Khosravan V (2002). Anticonvulsant effect of Crocus sativus L stigmas aqueous and ethanolic extracts in mice. Arch Iran Med.

[21] Karimi G, Hosseinzadeh H, Khaleghpanah P (2001). Study of antidepressant effect of aqueous and ethanolic of Crocus sativus in mice. Iran J Basic Med Sci.

[22] Hosseinzadeh H, Karimi Gh, Niapour M (2003). Antidepressant effect of Crocus sativus L. stigma extracts and their constituents, crocin and safranal in mice.

[23] Akhondzadeh S, Fallah-Pour H, Afkham K (2004). Comparison of Crocus sativus L and imipramine in the treatment of mild to moderate depression: A pilot double-blind randomized trial. BMC Complement Altern Med.

[24] Rios JL, Recio MC, Giner RM (1996). An update review of saffron and its active constituents. Phytother Res.

[25] World Health Organization (2010). WHO laboratory manual for the examination and processing of human semen.

[26] Wyrobek AJ, Gordon LA, Burkhart JG (1983). An evaluation of the mouse sperm morphology test and other sperm tests in nonhuman mammals: A report of the US environmental protection agency Gene-Tox program. Mutat Res.

[27] Hodjat M, Akhondi M, Amirjanati N (2008). The comparison of four different sperm chromatin assays and their correlation with semen parameters. Tehran Univ Med J.

[28] Talebi AR, Sarcheshmeh AA, Khalili MA (2011). Effects of ethanol consumption on chromatin condensation and DNA integrity of epididymal spermatozoa in rat. Alcohol.

[29] Sadeghi MR, Hodjat M, Lakpour N (2009). Effects of sperm chromatin integrity on fertilization rate and embryo quality following intracytoplasmic sperm injection. Avicenna J Med Biotechnol.

[30] Narayana K, D’Souza UJ, Seetharama Rao KP (2002). Ribavirin-induced sperm shape abnormalities in Wistar rat. Mutat Res.

[31] Xi L, Qian Z, Du P (2007). Pharmacokinetic properties of crocin (crocetin diagentiobiose ester) following oral administration in rats. Phytomedicine.

[32] Lear L, Nation RL, Stupans I (1992). Effects of cyclo-phosphamide and adriamycin on rat hepatic microsomal glucuronidation and lipid peroxidation. Biochem Pharmacol.

[33] Ordoudi SA, Befani CD, Nenadis N (2009). Further examination of antiradical properties of Crocus sativus stigmas extract rich in crocins. J Agric Food Chem.

[34] Aitken RJ, Clarkson JS, Fishel S (1989). Generation of reactive oxygen species, lipid peroxidation, and human sperm function. Boil Reprod.

[35] Iwasaki A, Gagnon C (1992). Formation of oxygen reactive species in spermatozoa of infertile patients. Fertil Steril.

[36] Lenzi A, Culasso F, Gandini L (1993). Placebo-controlled, double-blind, cross-over trial of glutathione therapy, in male infertility. Hum Reprod.

[37] Agarwal A, Ikemoto I, Loughlin KR (1994). Relationship of sperm parameters to levels of reactive oxygen species in semen spectimens. J urol.

[38] Armstrong JS, Rajasekaran M, Chamulitrat W (1999). Characterization of reactive oxygen species induced effects on human spermatozoa movement and energy metabolism. Free Radic Biol Med.

[39] Saleh RA, Agarwal A (2002). Oxidative stress and male infertility: From research bench to clinical practice. J Androl.

[40] Agarwal A, Saleh RA, Bedaiwy MA (2003). Role of reactive oxygen species in the pathophysiology of human reproduction. Fertil Steril.

[41] Gagnon C, de Lamirande E (1999). Male sterility and motility disorders.

[42] Gil-Guzman E, Ollero M, Lopez MC (2001). Differential production of reactive oxygen species by subsets of human spermatozoa at different stages of maturation. Hum Reprod.

[43] Suzuki N, Sofikitis N (1999). Protective effects of antioxidants on testicular functions of varicocelized rats. Yonago Acta Med.

[44] Huszar G, Sbracia M, Vigue L (1997). Sperm plasma membrane remodeling during spermiogenetic maturation in men: Relationship among plasma membrane beta 1,4-galactosyltransferase, cytoplasmic creatine phosphokinase, and creatine phosphokinase isoform ratios. Biol Reprod.

[45] Duru NK, Morshedi M, Oehninger S (2000). Effects of hydrogen peroxide on DNA and plasma membrane integrity of human spermatozoa. Fertil Steril.

[46] Qiu J, Hales BF, Robaire B (1995). Damage to rat spermatozoal DNA after chronic cyclophosphamide exposure. Biol Reprod.

[47] Codrington AM, Hales BF, Robaire B (2004). Spermiogenic germ cell phase-specific DNA damage following cyclophosphamide exposure. J Androl.

[48] Aziz N, Saleh RA, Sharma RK (2004). Novel association between sperm reactive oxygen species production, sperm morphological defects, and the sperm deformity index. Fertil Steril.

[49] Enciso M, Johnston SD, Gosálvez J (2011). Differential resistance of mammalian sperm chromatin to oxidative stress as assessed by a two-tailed comet assay. Reprod Fertil Dev.

